# Modified thoracoabdominal nerves block through perichondrial approach for laparoscopic cholecystectomy

**DOI:** 10.1590/1806-9282.20230962

**Published:** 2024-04-22

**Authors:** Ela Erten, Umut Kara, Fatih Şimşek, Muharrem Öztaş, Mehmet Anıl Süzer, Hasan Kamburoğlu, Mehmet Burak Eşkin, Serkan Şenkal, Ahmet Çoşar

**Affiliations:** 1 University of Health Sciences, Gulhane Training and Research Hospital, Department of Anesthesiology and Reanimation – Ankara, Turkey.; 2 Institute of Health Sciences, Gülhane Training and Research Hospital, Department of General Surgery – Ankara, Turkey.; 3 Private Çankaya Hospital, Department of Anesthesiology and Reanimation – Ankara, Turkey.; 4 Private Acıbadem Ataşehir Hospital, Department of Anesthesiology and Reanimation – Ankara, Turkey.

**Keywords:** Laparoscopic cholecystectomy, Nerve block, Postoperative pain, Anesthetics, local

## Abstract

**OBJECTIVE:**

A new block, namely, modified thoracoabdominal nerves block through perichondrial approach, is administered below the costal cartilage. We sought to compare the analgesic efficacy of the modified thoracoabdominal nerves block through perichondrial approach block with local anesthetic infiltration at the port sites in an adult population who underwent laparoscopic cholecystectomy.

**METHODS:**

Patients who will undergo laparoscopic cholecystectomy were randomized to receive bilateral ultrasound-guided modified thoracoabdominal nerves block through perichondrial approach blocks or local anesthetic infiltration at the port insertion sites. The primary outcome was the total amount of tramadol used in the first 12 h postoperatively. The secondary outcomes were total IV tramadol consumption for the first postoperative 24 h and visual analog scale scores.

**RESULTS:**

The modified thoracoabdominal nerves block through perichondrial approach group had significantly less tramadol use in the first 12 h postoperatively (p<0.001). The modified thoracoabdominal nerves block through perichondrial approach group's visual analog scale scores at rest (static) and with movement (dynamic) were significantly lower compared with the port infiltration group (p<0.05).

**CONCLUSION:**

Patients who received modified thoracoabdominal nerves block through perichondrial approach block had significantly less analgesic consumption and better pain scores than those who received port-site injections after laparoscopic cholecystectomy.

## INTRODUCTION

Laparoscopic cholecystectomy (LC) is less invasive and offers benefits compared with open surgery. However, postoperative pain remains a significant predictor of recovery^1,2^. Regional blocks such as TAP, erector spinae plane, rectus sheath, quadratus lumborum, and paravertebral blocks are commonly used in LC^3–5^. However, these blocks do not adequately block abdominal walls’ anterior and lateral parts and may cause sensory blockade of the surgical field^6,7^.

The thoracoabdominal nerves block through perichondrial approach (TAPA) is a new block that affects the thoracoabdominal nerves’ branches up to T5 in the cephalic direction and T1-L1 in the caudal direction^4,8^. In the TAPA block, LA is injected twice, at the costochondral corner, on the lower and upper surfaces of the chondrium. In contrast, modified TAPA (M-TAPA) only requires a single injection immediately below the costal cartilage^9^.

We sought to compare the analgesic efficacy of the M-TAPA block with that of LA infiltrations at the laparoscopic access sites in patients who underwent LC. The primary aim was to evaluate the total tramadol consumption via patient-controlled analgesia (PCA). The secondary aims were to compare postoperative visual analog scale (VAS) scores and frequency of rescue analgesic administration. We hypothesized that the M-TAPA block would offer improved pain management compared with port-site infiltration.

## METHODS

This study was carried out at a training hospital in Ankara, Turkey. The study was approved by the ethics committee of the Gülhane Training and Research Hospital (No. 2023/4). Our study was carried out in accordance with the provisions of the Consolidated Reporting Studies statement and the Declaration of Helsinki^10^.

According to the guidelines of American Society of Anesthesiology (ASA), patients with physical status I–II aged 18–65 years who will have elective LC were included. Before the study, patients were informed, and written consent was obtained. Patients with known allergies to any of the medications used in the study, local or systemic infection, a history of alcohol or drug abuse, inability to cooperate and understand the Turkish language, and patients with pregnancy were excluded. After obtaining a computer-generated randomization list consisting of eight blocks of 10 with an intergroup ratio of 1:1 and equally distributed in the two groups, 80 opaque envelopes with numbers 1–80 were sealed. The patients were randomly assigned M-TAPA group or port infiltration group.

All patients received general anesthesia with sevoflurane and remifentanil infusion. The surgery was performed using the standard four-trocar method. All patients received 1 g of paracetamol and 50 mg of dexketoprofen intravenously. Thirty minutes prior to emergence, all patients received 4 mg of ondansetron intravenously. At the end of the surgery, 2 mg/kg of sugammadex was used to reverse neuromuscular blockade.

### Modified thoracoabdominal nerves block through perichondrial approach block

**Figure 1:**
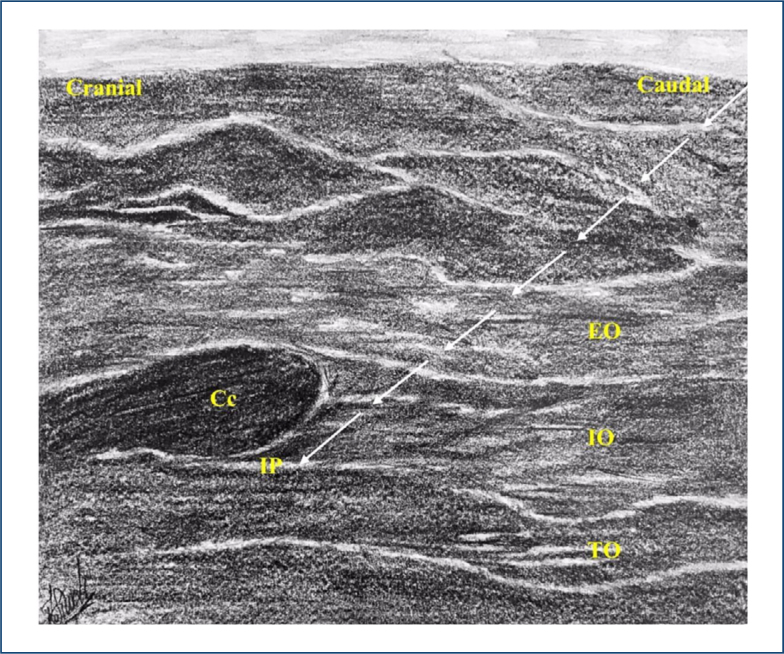
Modified thoracoabdominal nerves block through perichondrial approach block; reverse ultrasound anatomy. Cc: costal cartilage; EO: external oblique muscle; IO: internal oblique muscle; TO: transversus abdominis muscle; IP: injection point.

Ultrasound-guided M-TAPA block was administered bilaterally prior to emergence from general anesthesia after the surgical procedure by the same anesthesiologist. A linear high-frequency probe was used to identify the anatomy (Figure 1). At the level of the 10th rib in the midclavicular line, 20 mL of 0.25% bupivacaine was injected (arcus costarum) between the transversus abdominis muscle's upper fascia and the lower fascia of costochondral tissue. The same technique was followed on the other side.

### Port infiltration

The surgeon infiltrated 10 mL of 0.25% bupivacaine at each of the four laparoscopic port areas at the end of the surgery.

After the block and waking up from anesthesia, the patients were transported to the post-anesthesia care unit (PACU). Data collection was continued for 24 h in the ward. Rescue analgesia in the form of 75 mg of diclofenac was administered intramuscularly when the VAS score was >4. In case of nausea and vomiting in the postoperative period, 4 mg of ondansetron was administered as a rescue antiemetic.

Pain was measured using the VAS score at rest (static) and during movement (dynamic). It was rated from 0 to 10, from no pain to unbearable pain. The VAS scores were recorded postoperatively at PACU, 1, 2, 4, 8, 12, and 24 h. Postoperative complications related to the surgery such as nausea/vomiting, shoulder tip pain, or dizziness were evaluated. The Turkish version of the QoR-15 was used to evaluate the quality of recovery of the patients 24 h after surgery^11^.

The primary outcome of the study was the total amount of tramadol used in the first 12 h postoperatively. The secondary outcomes included the total IV tramadol consumption for the first postoperative 24 h (mg), the VAS scores at rest (in supine position) and with mobilization (described as transition from supine to sitting position), the QoR-15 score at 24 postoperative hours, rescue analgesic requirement (yes/no), incidence of postoperative complications (yes/no), incidence of nausea and vomiting (yes/no), length of stay (LOS) in PACU (min), and LOS at the hospital (days).

The retrospective data collected from patients undergoing LC showed that the tramadol consumption during the first 12 postoperative hours in the port infiltration group was 139±66 mg. We aimed to detect a reduction of at least 35% in tramadol consumption with a threshold of significance alpha=0.05 and a power of 90% as a result of the M-TAPA block. Therefore, the necessary sample size was 30 patients in each group. Sample size calculation was done using G-power^12^. After a dropout rate of 20%, 72 patients were enrolled in the study. Continuous variables are described as means±SD or median (Q1–Q3). We used the Shapiro-Wilk test to determine the normality of the data distribution. Normally distributed data were analyzed using an independent t-test, and non-normally distributed data were analyzed using the Mann-Whitney U test. Corrected confidence intervals and p-value were calculated by Bonferroni's method for multiple testing of repeated measurements. The effect size was calculated by dividing the absolute standardized test z-statistic by the square root of the number of pairs (n=60). A p<0.05 was considered statistically significant. Categorical variables were expressed as numbers (percentages), and their evaluations were made with Pearson's chi-square test. IBM SPSS Statistics (version 25.0, IBM Corp., Armonk, NY) was used for statistical analyses.

## RESULTS

A total of 73 patients were screened for eligibility between February and May 2023. We excluded six patients from the study because they did not meet the criteria, and four patients did not want to participate in the study. Notably, 63 patients were included in the study and randomized. One patient withdrew consent prior to anesthesia, and the surgical approach was changed from laparoscopic to open cholecystectomy in two patients. Thus, the study was completed by 60 patients without any adverse events or complications.

Data from 60 patients, including 31 patients from the M-TAPA group and 29 patients from the port infiltration group, were included in the analysis. The mean age of the patients was 49.26±12.16 years, and 44 (73.3%) patients were women. The mean surgical time was 95.08±26.83 min, and the mean total anesthesia time was 109.25±27.92 min. The median PACU-LOS was 30 min, and the median hospital LOS was 1 day in both groups. When the groups were compared in terms of these basic characteristics, they were found to be similar (p>0.05).

**Figure 2:**
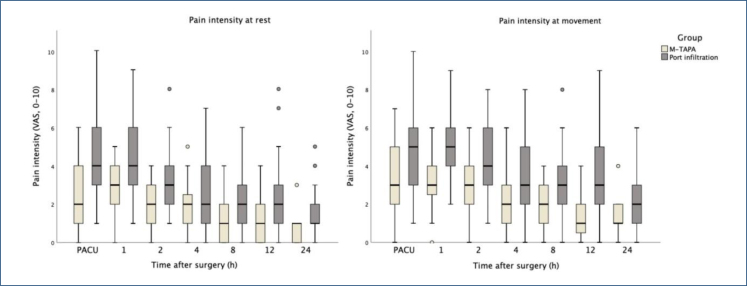
Comparisons of pain intensity at rest between the two groups. The box plot represents the median and interquartile range of the NRS in the modified thoracoabdominal nerve block through the perichondral approach and port infiltration groups during the study period. The upper lines represent the maximum value, whereas the lower lines represent the minimum value, excluding outliers. M-TAPA: modified thoracoabdominal nerve block through the perichondral approach; VAS: visual analog scale.

The following scores were significantly lower in the M-TAPA group than those in the port infiltration group: the postoperative pain VAS score at rest in the PACU and 1, 2, 12, and 24 postoperative hours and the postoperative pain VAS score during movement at all observation times (p<0.05) (Table 1). Figure 2 shows the comparisons of pain intensities between the two groups. The M-TAPA block reduced the mean tramadol requirements by 52.7% during the first 24 h compared with port-site infiltration.

**Table 1:** Comparison of pain scores, tramadol consumptions, and postoperative datas.

Variable	M-TAPA group (n=31)	Port infiltration group (n=29)	Effect size^a^ (95%^d^ or 99%^b^ CI)	Corrected^b^ p-value
PACU VAS-R*	2.45±1.72	4.27±2.46	-1.82^c^ (-2.91 to −0.73)^d^	0.001^b^
1st hour VAS-R	3.00 (2.00, 4.00)	4.00 (3.00, 6.00)	0.48 (0.000 to 0.000)^b^	<0.001^b^
2nd hour VAS-R	2.00 (1.00, 3.00)	3.00 (2.00, 4.00)	0.34 (0.007 to −0.012)^b^	0.009^b^
4th hour VAS-R	2.00 (1.00, 3.00)	2.00 (1.00, 4.00)	0.16 (0.195 to 0.216)^b^	0.206^b^
8th hour VAS-R	2.00 (0.00, 2.00)	2.00 (1.00, 3.00)	0.24 (0.051 to −0.063)^b^	0.057^b^
12th hour VAS-R	1.00 (0.00, 2.00)	2.00 (1.00, 3.50)	0.36 (0.003 to 0.006)^b^	0.004^b^
24th hour VAS-R	1.00 (0.00, 1.00)	1.00 (1.00, 2.00)	0.44 (0.000 to −0.001)^b^	0.001^b^
PACU VAS-M*	3.00 (2.00, 5.00)	5.00 (3.00, 6.50)	0.30 (0.013 to 0.020)^b^	0.017^b^
1st hour VAS-M	3.00 (2.00, 4.00)	5.00 (4.00, 6.00)	0.48 (0.000 to 0.000)^b^	<0.001^b^
2nd hour VAS-M	2.83±1.50	4.34±1.79	-1.50^c^ (-2.361 to 0.650)^d^	0.001
4th hour VAS-M	2.00 (1.00, 3.00)	3.00 (2.00, 5.00)	0.38 (0.002 to 0.005)^b^	0.004^b^
8th hour VAS-M	2.00 (1.00, 3.00)	3.00 (2.00, 4.00)	0.34 (0.004 to 0.008)^b^	0.006^b^
12th hour VAS-M	1.00 (0.00, 2.00)	3.00 (2.00, 5.00)	0.53 (0.000 to 0.000)^b^	<0.001^b^
24th hour VAS-M	1.00 (1.00, 2.00)	2.00 (1.00, 3.00)	0.39 (0.001 to 0.004)^b^	0.003^b^
2nd hour tramadol consumptions (mg)	24.61±17.25	40.00±18.65	-15.38 (-25.59 to −5.17)^e^	0.004^a^
4th hour tramadol consumptions (mg)	42.30±22.85	84.16±31.74	-41.85 (-57.49 to −26.21)^e^	<0.001^a^
8th hour tramadol consumptions (mg)	56.92±30.82	125.83±54.20	-68.91 (-93.73 to −44.08)^e^	<0.001^a^
12th hour tramadol consumptions (mg)	73.84±42.99	169.16±73.42	-95.32 (-129.21 to −61.42)^e^	<0.001^a^
24th hour tramadol consumptions (mg)	86.15±59.53	200.00±85.26	-113.84 (-155.39 to −72.29)^e^	<0.001^a^
24th hour QoR-15 score	110.77±19.13	98.48±18.09	—	0.013
Rescue analgesic requirement (Yes:No)	1:30 (3.2%:96.8%)	12:17 (41.4%:58.6%)	—	<0.001
Postoperative complications (Yes:No)	12:19 (38.7%:61.3%)	19:10 (65.5%:34.5%)	—	0.038
Nausea/vomiting (Yes:No)	10:21 (32.3%:67.7%)	18:11 (62.1%:37.8%)	—	0.021

Values are presented as mean±standart deviation, median (Q1, Q3) or frequency (%). VAS-R: visual analog scale score at rest; M-TAPA: modified thoracoabdominal nerves block through perichondrial approach; PACU: post-anesthesia care unit; VAS-M: visual analogue scale score at movement; QoR: quality of recovery.

a Effect size is calculated by dividing the absolute standardized test statistic z by the square root of the number of pairs (n=60).

b 99%CI and corrected p-value were calculated by Bonferroni's correction for multiple testing of repeated measurement.

c The mean difference was used instead of the effect size.

d 95%CI was calculated.

e 95%CI and corrected p-value were calculated by Bonferroni's correction for multiple testing of repeated measurement.

* Measured immediately after PACU arrival. p<0.05 is considered statistically significant.

## DISCUSSION

We compared the analgesic efficacy of LA infiltration at the port-insertion sites with that of ultrasound-guided M-TAPA block in patients undergoing LC. We observed that patients who received M-TAPA block had significantly lower tramadol consumption and better VAS scores than patients who received port-site infiltrations.

The sources of pain after LC are visceral and somatic pain. Somatic pain is caused by the port-site incisions, whereas visceral pain is caused by the peritoneal stretch and manipulation of the abdominal tissues^1^. Ultrasound-guided interfascial plane blocks offer varying effective analgesia for abdominal surgeries compared with neuraxial blocks^5^. Serratus intercostal plane (SIP) and oblique subcostal transversus abdominis plane (OSTAP) blocks affect anterior dermatomes, and quadratus lumborum and erector spinae plane blocks affect the posterior dermatomes.

The TAPA block has analgesic effects by blocking the anterior and lateral parts of the thoracoabdominal wall. The anterolateral abdominal wall innervation is done by T7-L1 nerves anterior branches^13^. Intercostal nerves pass under the chondrium and connect the origin of the transversus abdominis muscle to the cartilage^14^. In the M-TAPA block, LA is injected under the costal cartilage at the junction of the transversus abdominis muscle with the 10th rib and blocks the anterolateral cutaneous branches of the T5–T12/L1 thoracoabdominal nerves^15^. Ohgoshi et al., demonstrated that the M-TAPA block affects anterior branches of T6–T12 thoracoabdominal nerves^16^, whereas Bahadır et al., reported an effect in a wide dermatome area extending from T4 to T11–T12^17^.

Several previous studies have evaluated the effects of interfacial plane blocks on pain after LC. Saxena et al., compared ultrasound-guided abdominal field block with port-site infiltrations and reported that abdominal field block provided superior analgesia after LC^18^. Molfino et al., compared TAP block with port-site infiltrations after LC and reported that both groups had similar analgesic effectiveness^19^. In contrast to the results obtained in our study, port-site infiltration and TAP block may have similar analgesic effects since the TAP block can be effective in the T7–12 dermatome region. The procedures on the supraumbilical area may involve the use of the interfacial plane or SIP blocks^6,20^. The M-TAPA block should be preferred over these blocks in LC because, to reduce the pain at the incision sites in LC, the anterior and lateral branches of the intercostal nerves must be blocked same time. However, the anterior and lateral cutaneous branches cannot be blocked with the SIP and OSTAP blocks, respectively^6,7^. The literature review revealed that three studies have evaluated the effects of the M-TAPA block on post-LC pain. Gungor et al., compared the M-TAPA block with LA infiltration after LC and reported that, in the M-TAPA group, postoperative pain and the need for rescue analgesia were significantly lower^21^. Bilge et al., showed that the M-TAPA block decreased pain and consumption of tramadol compared with the control group^22^. Erturk et al., compared the TAPA block with the M-TAPA block after LC and showed that NRS scores, tramadol consumption, and rescue analgesic use were similar in the postoperative period^4^. Our results are consistent with the results of these three studies that the M-TAPA block prevents post-LC pain.

Another issue is the evaluation of the quality of postoperative recovery from the patient's perspective. Gungor et al., evaluated patient satisfaction during the postoperative period and reported that the M-TAPA group's scores were better^21^. Bilge et al., also found better QoR scores in the M-TAPA group^22^.

The study has a few limitations. First, the postoperative pain was evaluated for 24 h only. The analgesic effect of a single-shot interfascial plane block persists for 24–48 h^5^. Second, the dermatome field affected by the M-TAPA block or port-site infiltration was not examined to evaluate the actual effects of the blocks. Finally, a fixed volume of LA was used for the block.

## CONCLUSION

Compared with port-site infiltrations, the M-TAPA block has resulted in a significant decrease in the systemic analgesic demand following LC. Furthermore, considering the dermatome areas impacted by the M-TAPA block, further research should be conducted on its usefulness in open surgical procedures.
